# Supplementary figures and images for: Repeat Ascaris challenge reduces worm intensity through gastric cellular reprograming
Source: PLoS Negl Trop Dis. 2025 May 30;19(5):e0013141. doi: 10.1371/journal.pntd.0013141 (PMC12151467; doi:10.1371/journal.pntd.0013141)

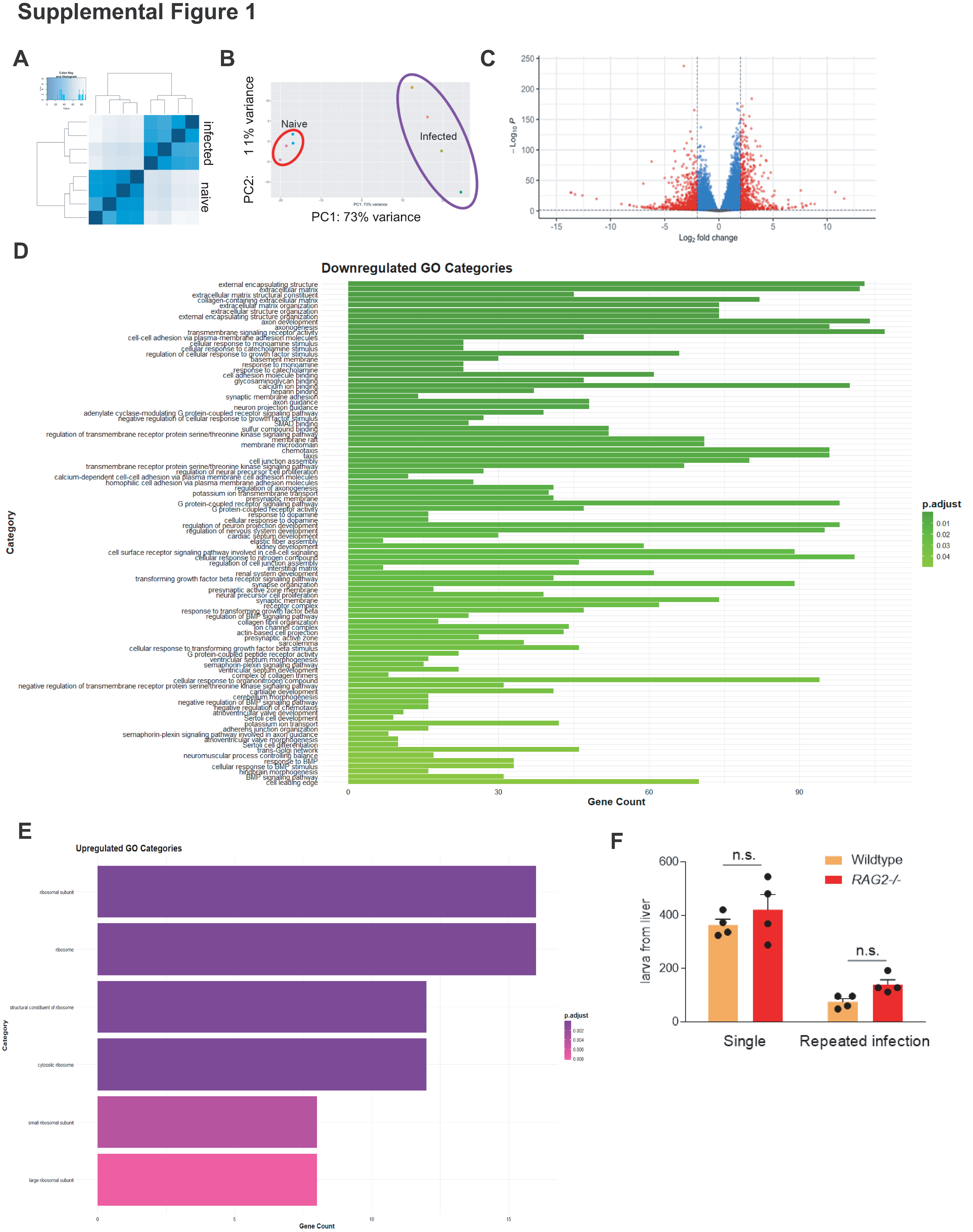

Supplement: S1 Fig — Heat map (A) and principal component analyses (B) demonstrate stark variations in transcriptomes based on infection status. Volcano plot (C) shows significantly differentially expressed genes (|log2 fold change| > 2 and <0.05 p adjusted value) in the gastric mucosa of mice repeatedly challenged with Ascaris compared to naïve mice. Gene ontology (GO) analysis illustrate (D) significantly downregulated and (E) upregulated pathways in infected gastric tissue compared to naive tissue. (F) There was no difference in Ascaris larvae intensity in the liver between wild-type and RAG2-/- mice following single and repeat Ascaris infection. (n = 4, mean±S.E.M, n.s.: not significant, using two-way ANOVA. Data are shown as representative of two independent experiments.). (TIF) [file pntd.0013141.s001.tif]
