# Supplementary material for: Repeat Ascaris challenge reduces worm intensity through gastric cellular reprograming
Source: PLoS Negl Trop Dis. 2025 May 30;19(5):e0013141. doi: 10.1371/journal.pntd.0013141 (PMC12151467; doi:10.1371/journal.pntd.0013141)
Supplement: Attachment — Submitted filename: Response to reviewers 03-06-25.docx [file pntd.0013141.s003.docx]

**Response to Reviewers:**

We appreciate the comments and feedback from the reviewers. We have taken the comments into consideration and made the appropriate updates per the reviewers comments.

**Reviewers' Comments:**

**Reviewer #1:**

1. Abstract Evaluation:
Assess the clarity and conciseness of the abstract to ensure it covers the key points and objectives of the study.
Check for proper use of abbreviations; specifically, confirm that each abbreviation is accompanied by the full term upon first use.

**Author response:** *We have assessed the clarity and conciseness of the abstract as suggested by the reviewer*

2. Introduction Review:
Evaluate the structure and logic of the introduction to ensure it effectively introduces the topic, highlights the research’s significance, and provides a brief review of previous studies.
Verify the accurate and appropriate use of scientific references to support the arguments made.
Suggest adding studies that discuss the roles of microbiome and gastric cells in reducing infection intensity to reinforce the article’s arguments.

**Author response:** *We have assessed the structure and logic of the introduction as suggested by the reviewer. We opted to not include studies on microbiome and gastric cells as this is outside the scope of this manuscript.*

3. Materials and Methods Review:
Evaluate the accuracy and clarity of the methods to ensure reproducibility.
Assess the control variables and provide a thorough justification for selecting specific doses and the number of experimental challenges for the mice.
Ensure the correct selection and description of statistical methods for data analysis, and suggest applying more precise statistical tests if needed.

**Author response:** *We have assessed the clarity of the accuracy and clarity of the methods suggested by the reviewer*

**Reviewer #2:** The authors clearly articulate their hypothesis and provide a good introduction to set the scene so that the reader can follow. However, I feel that the study design does not address the stated objectives that aimed to reveal age-dependent effects of *Ascaris* infection by using repeated infection mouse model. Please see further details in the "Summary and General comments".

Methodology section should be expanded to ensure readers understand exactly how the research was performed and to allow data reproducibility. For example, please expand:

1) qPCR method description to include how was the RNA extracted, the primer sequences used, which gene was used to calculate relative expression and how was relative expression calculated.

**Author response:** *We have clarified in the methods that RNA extraction was performed using the TRIzol manufacturer's protocol. For qPCR, we used commercially available TaqMan probes rather than primers, as noted in the respective section within the methods. Additionally, we have specified that relative expression calculations were based on the ΔΔCt method.*

2) western blotting method description to include how was the band intensity quantified and normalized, and which secondary antibodies were used.

**Author response:** *As suggested by the reviewer we have included details on the secondary antibody as well as band quantification in the respective section.*

3) histopathology, could you clarify if the 4 sections are from 1 mouse?

**Author response:** *We apologize for the confusion in the histopathology sections. The four sections were obtained from four individual mice. We have clarified this in the Methods section.*

4) Statistical analysis description - it would also be beneficial to the manuscript and readers to define which experiments were performed in duplicate and if those were technical or biological replicates.

**Author response:** *We apologize for the confusion. We have revised the statistic section to clarify experiment designs.*
The references cited in the RNAseq method description do not match references in bibliography

**Author response:** *We have revised the RNAseq references.*

Flow cytometry method description should be revised as it currently includes description of analysis that is not shown in the manuscript (eg. T-bet/GATA/etc).

**Author response:** *We have clarified the flow cytometry section and removed unnecessary descriptive analysis.*

Additionally, I note the data will be made publicly available, however, the github link takes reader to "Page not found" and PRJNA1141177 could not be located on SRA or BioProject servers potentially due to paper being currently under review.

**Author response:** *We apologize for this error.* *We have amended the link in the text.*

**Results**
-Does the analysis presented match the analysis plan?
-Are the results clearly and completely presented?
-Are the figures (Tables, Images) of sufficient quality for clarity?

**Reviewer #1**: Results Analysis:
Evaluate the clarity and accuracy in presenting the results and ensure alignment with the study’s objectives.
Review the adequacy and appropriateness of tables and figures for displaying data.
Assess the statistical analysis of the results to confirm the validity and accuracy of the conclusions and suggest additional statistical analyses if necessary.

**Author response:** *We have assessed the clarity of the accuracy and clarity in the results and ensured alignment with the study’s objectives suggested by the reviewer*

**Reviewer #2:** The authors present the data clearly and provide nice schematics of the mouse infection schedules.

For further clarity could the authors add the following details to the Results Section 1/Fig. 1:
1) define "o.g." in the legend
2) expand legend to include what the protein levels of Atp4a and AMCase are normalised to

**Author response:** *We have defined "o.g." as oral gavage and provided additional details on Western blot normalization in the legend of Fig 1.*

For further clarity could the authors add the following details to the Results Section 2:
1) add p-value and logFC cut-offs for volcano plot
2) add what the mRNA levels are relative to in Fig. 2B)
3) if RAG2-/- mice were used as per methods please change labels on figures and throughout the manuscript
4) for the observation “Larva burden in RAG-/-, repeat Ascaris challenge mice were similar to wild-type, repeat Ascaris challenge mice (Figure 1B)" please graph side-by-side and perform statistical analysis with select comparisons of Single infection in RAG-/- vs WT mice, and Repeat Infection in RAG-/- vs WT mice.

**Author response:** *We have included p-values and cut-offs for volcano plot and mRNA level relativity in the figure legend in Fig 2B. RAG2^-/-^ mice were only used once as depicted in Figure 2D. All other experiments were done using wild-type mice. As suggested by the reviewer we have provided the side-by-side graph and statistical analysis from single infection WT vs RAG-/- in Supplemental Figure 1F*

For further clarity could the authors add the following details to the Results Section 3:
1) please label the pathways that the genes are associated with on the figures (Fig 3A+B). It would be beneficial to mention that upregulation of eg. muc1 is associated with gastrointestinal worm infections. It would also be beneficial to show a pathway enrichment analysis of the RNAseq data
2) Fig 3 legend - D) is mislabeled to F)
3) Scale bar on histopathology images are difficult to see. I would recommend to add these details in the figure legend, including the scale bar on the insert image.

***Author response:*** *As suggested by the reviewer we have added the pathways at the top of Fig 3A and 3B and included pathway enrichment analysis in the supplemental figure 1 (D-E). We have corrected the Fig 3 legend D and F and have we added a more clear scale bar to the histopathtology images as recommended by the reviewer.*

In sections 2 and 3, I feel that comparing repeatedly infected mice to naïve mice is expected to show huge differences in gene expression between infected and uninfected animals. This is also clear from the PCA plot where the presence/absence of infection accounts for most of the variance observed, and also from the genes identified as upregulated (muc1, ttf2). However, it is not mentioned in the manuscript that these changes were expected.

***Author response:*** *While we observed a unique gastric transcriptome in repeat Ascaris infection compared to naïve mice, upregulated and downregulated genes related to gastric cellular changes and pyloric metaplasia was hypothesized but not expected.* *However, this work in sections 2 and 3 was concordant with our prior qPCR data as well. We have added commentary through section 2 and 3 to support the link between the transcriptomic data and the qPCR data that was previously identified.*

**Conclusions**

-Are the conclusions supported by the data presented?
-Are the limitations of analysis clearly described?
-Do the authors discuss how these data can be helpful to advance our understanding of the topic under study?
-Is public health relevance addressed?

**Reviewer #1**: Discussion and Conclusion Review:
Evaluate the interpretation of results and comparison with prior studies to strengthen the arguments.
Assess the logical flow and coherence of the conclusions, including a clear mention of the study’s limitations.
Recommend exploring the potential for generalizing the results to humans and addressing future experimental needs on the topic.

**Author response:** *As suggested by the reviewer we have evaluated the interpretation of results and comparison with prior studies. We have also included study limitations and written a statement on generalizing the results to humans.*

**Reviewer #2**: I feel that some of the conclusions are not supported by the data presented here as expanded in the "Summary and General Comments" section.

It would be beneficial to expand on the limitations of the analysis performed and on the significance of the H. pylori infections.

**Author response**: *As suggested by the reviewer a discussion on study limitations has been included in the conclusions. We have also expanded on the significance of these findings on* H.pylori *infection.*

The authors discuss how these data can advance our understanding of the topic under study and propose that the new pathway they identified in repeat Ascaris infection could be a vaccine target for other gastrointestinal nematodes. Could the authors please provide appropriate references for statements regarding the need for vaccine development.

**Author response**: *We have now included references regarding the need for vaccine development.*

Additionally, the authors make interesting links to H. pylori infection which is also of public health relevance.

Please revise reference for the following sentence: “Adherence to the gastric corpus epithelium allows H. pylori colonization and expansion of pyloric metaplasia in the gastric corpus in a positive feedback loop8.” – reference 8 does not mention H. pylori.

**Author response:** *We would like to thank the reviewer for identifying this error. We have corrected the reference.*

Please amend statement “However, no studies to date have examined co-infection.” as this in now out of date – see publication <https://doi.org/10.1016/j.parint.2024.102896>

**Author response:** *We appreciate the reviewers suggestion. As the reviewer highlights, co-infection does exist however, the mechanisms that lead to coinfection have not been explored. We have clarified this statement and included the reference suggested by the reviewer.*

**Editorial and Data Presentation Modifications?**

Use this section for editorial suggestions as well as relatively minor modifications of existing data that would enhance clarity. If the only modifications needed are minor and/or editorial, you may wish to recommend “Minor Revision” or “Accept”.

**Reviewer #1**: Scientific and Quality Review of Figures and Tables:
Figures and tables convey scientific information effectively and are generally suitable for publication. However, Figure D has low quality and requires revision to enhance clarity for publication.
Ensure that all abbreviations and symbols are clearly explained in the images.

**Author response:** *As suggested by the reviewer we have updated figures to enhance the clarity for publication and we have ensured all abbreviations and symbols are explained.*

**Reviewer #2:** Overall the manuscript is very well presented, with some small editorial revisions:

1) Replace "," with "." in line: "Based on decreased chia1 mRNA expression we hypothesized that chief cells were undergoing transdifferentiation, Indeed, stomach transcriptomic data demonstrated increased gene expression of trefoil factor 2 (ttf2) also known as spasmolytic polypeptide and muc1 as well as decreased gene expression of mist1 (Figure 3A) following repeat Ascaris challenge."
2) Replace "follow" with "following" in line: “Gastric CD4+ T cells are decreased follow repeat Ascaris challenge”.
3) Replace RAG-/- label to RAG2-/- as mention before
4) Scale bars size as mentioned before

**Author response:** *We appreciate the reviewers comments and have made the recommended modifications.*

**Summary and General Comments**

Use this section to provide overall comments, discuss strengths/weaknesses of the study, novelty, significance, general execution and scholarship. You may also include additional comments for the author, including concerns about dual publication, research ethics, or publication ethics. If requesting major revision, please articulate the new experiments that are needed.

**Reviewer #1**: 1. Scientific Originality Check:
Assess the potential for similarity or overlap with previous studies to ensure scientific originality.
If highly similar or even identical studies are found, they will be highlighted and recommended for comparison.
2. Review of Abbreviations and Scientific Terms:
Ensure correct usage of abbreviations with full terms introduced at first use.
Certain abbreviations like "AMCase" and "Ascaris suum" were not introduced with full terms initially and require correction.
3. Italicization of Genus and Species Names:
Confirm that genus and species names are correctly italicized. This should be corrected in cases like Ascaris suum and Helicobacter pylori.
4. Reference Review According to PLOS Neglected Tropical Diseases Guidelines:
Align the references with the journal’s specific formatting guidelines, which require full author names, year of publication, full article title, italicized journal name, volume, and page numbers. For example:
Reference 2 should have a complete article title, and the journal name should be italicized.
Reference 10 should follow the standard format without quotation marks around the article title, and the author order should be corrected.
Reference 21 incorrectly uses numerals instead of the full journal name, which needs correction.
5. English Language and Writing Quality Assessment:
The article’s English language is fluent and academic, yet certain sentences need rewriting for improved clarity and precision. Additionally, minor grammatical errors require correction. For example:
In the sentence "Following oral ingestion of Ascaris eggs, larvae use AMCase secreted by gastric chief cells...", rewriting as "After ingestion of Ascaris eggs, larvae utilize AMCase, which is secreted by gastric chief cells, to hatch" enhances clarity.
In "We found that repeat Ascaris challenge caused cellular changes in the gastric mucosa which reduced worm intensity in the liver", using "that" instead of "which" improves scientific accuracy.
In "Once hatched, larvae translocate across the gastric mucosa to initiate the larval migratory cycle", changing "Once hatched" to "After hatching" eliminates minor grammatical issues.

**Author response:** *We have assessed the scientific originality of the presented study, reviewed abbreviations and scientific terms and have made corrections in the manuscript.* *Genus and Species names are italicized, and we have aligned the references with the journals formatting guidelines. Additional grammatical errors have been corrected.*

**Reviewer #2:** The authors aim to demonstrate age-dependent effects of Ascaris infection are due to cellular changes in the gastric mucosa barrier. However, I feel that the data do not fully support this conclusion. Specifically, the authors compare gene expression changes (by RNAseq, qPCR and western blot), histopathological changes and the number of CD4+ T cells in mice repeatedly infected with Ascaris against naïve (uninfected) mice. The basis of the research is there, however, this study requires additional experiments. Particularly, I feel that inclusion of single Ascaris infection in their RNAseq, qPCR, western blot, histopathology and Flow Cytometry experiments (compared to the repeat infection) is required in order to make conclusions specifically on age-dependent effects. Inclusion of singly-infected WT mice was already used in the manuscript to clearly show that repeated infection leads to lower worm burdens, therefore if tissues have been saved from those singly-infected mice they could be used to provide the additional data requested. Alternatively, the authors should include more information that clarifies and justifies their choice of comparing repeated infection to uninfected mice only and revise manuscript conclusions to reflect that the cellular changes observed are not specifically an age-dependent effect and that these changes could be present in single infection. To continue with, the authors clearly show using a RAG-/- mouse model that adaptive immune responses are not involved in the reduction of worm burdens observed in repeat infection vs single infection. It would be interesting (although not necessary for the revisions asked here) to investigate the differences in gene expression between the repeat and single infection in the RAG-/- model as it could reveal new or provide additional evidence supporting the authors' hypothesis.

Other comments:
1) Affiliation no. 4 is not attributed to any author.
2) Please define AMCase in the abstract and ensure all species names are in italics (line 12)
3) It would be beneficial to the reader to expand on why changes to immunity do not fully explain age-dependent intensity changes - line "While each of these mechanisms are likely contributing to age-dependent Ascaris worm intensity, none fully explain observed age-dependent intensity changes."
4) Please cite appropriate literature for line "We have previously shown…cells to hatch."
4) The authors should revise the references and ensure correct citation. For example, reference 1 and 7 the link takes reader to “Page not found” while references 8, 11, 13, 15 require further attention.

In general, in the future it would be beneficial to adapt manuscript and format it according to the author guidelines of the journal chosen for submission. For example, an author summary is required but currently not available, the funding sources should be removed from the acknowledgements, pages and lines are not numbered etc.

**Author response:** *We agree with the reviewer that including single infection to compare gene expression, histopathological changes and immune responses to the repeat infection model would enhance this manuscript. Unfortunately we do not have the ability to do this single infection currently nor do we have saved tissue from the previous experiment. This has been included in our limitations in the manuscript. We are currently building a chronic re-infection model that we will compare to single infection and the model that was used in this manuscript in the future as we acknowledge the importance of this information. We have made the additional changes suggested by the reviewer including defining AMCase, species name in italics and the references have been updated. We want to thank this reviewer for their in-depth analysis of this paper and very thorough feedback that helped the authors make significant revisions and updates to the manuscript. The feedback was very appreciated and helped strengthen the paper.*
