# Supplementary material for: Repeat Ascaris challenge reduces worm intensity through gastric cellular reprograming
Source: PLoS Negl Trop Dis. 2025 May 30;19(5):e0013141. doi: 10.1371/journal.pntd.0013141 (PMC12151467; doi:10.1371/journal.pntd.0013141)
Supplement: Attachment — Submitted filename: 20250512_responsetoreviewers_JWedits.docx [file pntd.0013141.s004.docx]

**Response to Reviewers**

We appreciate the comments and feedback from the reviewers. We have made the appropriate updates per the reviewers’ comments.

**Reviewers’ Comments**

Methods

**Reviewer #1:** *Are the objectives of the study clearly articulated with a clear testable hypothesis stated?*

Yes, the objectives of the study are clearly articulated, and the testable hypothesis regarding the role of gastric cellular reprogramming in reducing Ascaris worm intensity after repeated infections is stated clearly. The study aims to explore whether gastric cellular adaptations, such as pyloric metaplasia, contribute to reduced worm burden during repeated Ascaris infection.

**Author response:** Thank you for this comment.

*Is the study design appropriate to address the stated objectives?*

Yes, the study design is appropriate. The use of a repeated Ascaris challenge model in mice allows for the investigation of the cellular and immune changes induced by recurrent infections, and the results support the hypothesis that gastric cellular changes are key in modulating infection intensity.

**Author response:** Thank you for this feedback.

*Is the population clearly described and appropriate for the hypothesis being tested?*

Yes, the study clearly describes the mouse model used, particularly the use of wild-type and RAG2-/- mice. The mouse population used is appropriate for testing the hypothesis, as the model simulates human Ascaris infection and is suitable for studying immune responses and gastric cellular changes.

**Author response:** Thank you for this comment.

*Is the sample size sufficient to ensure adequate power to address the hypothesis being tested?*

The sample size appears to be adequate for the comparisons made (n ≥ 4). However, more details could be provided on how power was calculated to justify the chosen sample size, especially for key comparisons.

**Author response:** We thank the reviewer for this thoughtful comment. Sample size was determined based on preliminary data and was powered to detect a minimum effect size of 1.79-fold with 80% power at a significance level (α) of 0.05. These parameters guided our choice of using n ≥ 4 group. We have added this justification to the Methods section (Statistical analysis) of the revised manuscript

*Were correct statistical analysis used to support conclusions?*

Yes, correct statistical analyses, such as two-tailed Student’s t-tests and ANOVA, were used to support the conclusions. The inclusion of p-values and fold changes is appropriate for the analysis.

**Author response:** Thank you for this feedback.

*Are there concerns about ethical or regulatory requirements being met?*

The ethical statement is included, and all experimental protocols were approved by the Institutional Animal Care and Use Committee. Based on the information provided, there are no concerns regarding ethical or regulatory requirements.

**Author response:** Thank you for your comments.

**Reviewer #2:** (No Response)

Results

**Reviewer #1:** *Does the analysis presented match the analysis plan?*

Yes, the analysis matches the plan described. The study adequately addresses the effect of repeated Ascaris infections on gastric mucosa, including the impact on worm intensity and gastric cellular changes.

**Author response:** Thank you for this comment.

*Are the results clearly and completely presented?*

Yes, the results are presented clearly. Data are shown with appropriate statistical analysis, and the figures help to elucidate the findings. However, the figure legends could benefit from more detailed descriptions of what the data represent (e.g., relative gene expression normalization).

**Author response:** We appreciate this feedback and agree that the figure legends would be strengthened with more detailed descriptions of the data. We have included more information in all figure legends as requested by the reviewer.

*Are the figures (Tables, Images) of sufficient quality for clarity?*

Overall, the figures and images are of sufficient quality. However, there are a few suggestions for improvement:

The histopathology images could have clearer scale bars and more detailed legends, including magnifications and methods of quantification.

**Author response:** We have integrated a new scale bar in Fig 3 and included more details regarding scale bars and staining in the legend.

In Figure 2, the volcano plot could benefit from a clearer explanation of the p-value and fold change cut-offs.

**Author response:** We have included more details regarding the volcano plot in supplemental figure legend.

**Reviewer #2:** (No Response)

Conclusions

**Reviewer #1:** *Are the conclusions supported by the data presented?*

Yes, the conclusions are supported by the data. The results from the repeated Ascaris challenge model show that gastric cellular changes, such as pyloric metaplasia, correlate with a reduction in worm intensity, which aligns with the hypothesis that gastric mucosal adaptations contribute to altered infection intensity.

**Author response:** Thank you for your feedback.

*Are the limitations of analysis clearly described?*

The limitations could be more explicitly discussed, particularly regarding the use of a short-term infection model (2 weeks) and how this might limit generalization to long-term, chronic infections. More discussion on how the findings may differ in humans or in a long-term setting would strengthen the conclusions.

**Author response:** We appreciate this feedback and agree that our discussion would be improved with a more robust discussion regarding the relevance of these findings in a short-term model for human health. These changes are reflected in the discussion section.

*Do the authors discuss how these data can be helpful to advance our understanding of the topic under study?*

Yes, the authors effectively discuss how their findings contribute to our understanding of the mechanisms behind age-dependent changes in Ascaris worm intensity, particularly the role of gastric cellular adaptations. They suggest potential applications in the development of therapeutic interventions or vaccines.

**Author response:** Thank you for your comments.

*Is public health relevance addressed?*

The study addresses public health relevance, especially in terms of understanding the factors that influence worm burden and potential implications for vaccine development in Ascaris-endemic regions.

**Author response:** Thank you for this feedback.

**Reviewer #2:** (No Response)

Editorial and Data Presentation Modifications

**Reviewer #1:** Editorial suggestions and minor modifications:

Clarify the normalization method used for qPCR data in the figures and legends (e.g., relative to control group).

**Author response:** Thank you for this recommendation. We have amended the legends for figures 1-3:

Improve figure legends, especially in Figure 2, to include more detail on statistical cut-offs and p-values.

**Author response:** We have included more details regarding the volcano plot in supplemental figure legend.

Add clearer labels and scale bars to histopathology images in Figure 3.

**Author response:** We have replaced new scale bars in Fig 3 and included more details regarding scale bars and staining in the legend.

Consider providing more details on the pathway analysis from the RNA sequencing data.

Ensure that the references for RNA sequencing methods are updated and match the bibliography.

**Author response:** Details regarding volcano plots are updated in the Method section and figure legends. References have been updated.

**Reviewer #2:** There are some spelling mistakes, missing words, and lack of spaces between words across the document. Some examples include:

- “Gastric mucosal injury triggers the development of gastric ell reprogramming…” - ell=cell?

- “Bulk RNA sequencing: Stomach tissues from mice were collected 18 days after the initial A. suum, which was 4 days after the last infection…” - missing the word infection after the A. suum?

**Author response:** Thank you for this feedback. Spelling mistakes, missing words, and lack of spaces have been corrected in lines 37, 150, 172, 182, 198, 213, 215, 228, 230, 297, and 325.

Summary and General Comments

**Reviewer #1:** Strengths: The study is well-designed and provides valuable insights into the role of gastric mucosal adaptations in modulating worm intensity following repeated Ascaris infection. The methodology is appropriate, and the results are clearly presented, supporting the hypothesis.

**Author response:** Thank you for your comments.

Weaknesses: The limitations of the study could be discussed more thoroughly, especially regarding the short duration of the infection model. Additionally, more detailed information on the statistical analyses, including power calculations and sample size justification, would enhance the rigor of the study.

**Author response:** We appreciate and agree with these suggestions. We have included more thorough discussion of the limitations and relevance of our short-term model. These changes are reflected in lines 221-233 and are excerpted below:

We have also included more detailed information on the statistical analyses which are copied below:

Novelty and Significance: The study is novel in its focus on gastric cellular reprogramming, particularly pyloric metaplasia, as a mechanism of reduced worm intensity following repeated Ascaris infection. The findings could potentially inform vaccine development and public health strategies in endemic regions.

**Author response:** Thank you for your feedback.

Suggestions for Major Revision: No new experiments are necessary, but more clarification of the methods and data presentation, as well as additional discussion on the limitations and relevance to human disease, would strengthen the manuscript.

**Author response:** Thank you for these comments. We agree that both increased clarification of methods and data presentation and additional discussions of limitations and relevance have strengthened our manuscript. We have incorporated the following changes in response to your suggestions:

**Reviewer #2:** The authors have taken several of the reviewers suggestions on board and the manuscript has now a strong discussion section. In particular, the authors expand on the implications of *H. pylori* co-infection and acknowledge in the discussion the need for further experiments/comparisons with single infections too, and with models across varying ages to be able to comment on age-dependent effects.

**Author response:** Thank you for your comments.

Most (if not all) of the references require further attention. To point some of issues with references, many only report the first author without the "et al"; reference 44 is missing the volume(issue)pages information, etc.

Furthermore, as mentioned previously, references 1 and 7 links take reader to "Page not found". Please update these references with the appropriate links to the latest IHME and CDC data.

**Author response:** We have corrected all references and updated URL links.
